# Karyotype morphology suggests that the *Nyctibius griseus* (Gmelin, 1789) carries an ancestral ZW-chromosome pair to the order Caprimulgiformes (Aves)

**DOI:** 10.3897/CompCytogen.v6i4.3422

**Published:** 2012-11-30

**Authors:** Leonardo Martin Nieto, Rafael Kretschmer, Mario Angel Ledesma, Analía Del Valle Garnero, Ricardo José Gunski

**Affiliations:** 1 Dom Bosco Catholic University (UCDB), Campo Grande, Mato Grosso do Sul, Brasil; 2 Federal University of Pampa (UNIPAMPA) Campus São Gabriel, RS, Brasil; 3 Genetics Laboratory – El Puma Ecological Park – Ruta 12, Km. 12,5 – Candelaria, Misiones, Argentina

**Keywords:** C and Ag-NOR bands, macrochromosomes, microchromosomes, ZW sex chromosomes, new karyotype

## Abstract

Studies of karyotypes have been revealing important information on the taxonomic relationships and evolutionary patterns in various groups of birds. However, the order Caprimulgiformes is one of the least known in terms of its cytotaxonomy. So far, there are no cytogenetic data in the literature on birds belonging to 3 of 5 families of this order -Nyctibiidae, Steatornithidae and Aegothelidae. For this reason, the aim of our study was to describe the karyotype of *Nyctibius griseus* (Gmelin, 1789) (Aves, Nyctibiidae, Caprimulgiformes) and contribute with new data that could help to clarify the evolutionary relationships in this group. Bone marrow was cultured directly to obtain material for the chromosome study. C-banding was used to visualize the constitutive heterochromatin and Ag-NOR-banding to reveal nucleolus organizer regions. The diploid number observed was 2n=86±. Using sequential Giemsa/C-banding staining, we determined that the W chromosome was entirely C-band positive with the two most prominent markers in the interstitial and distal regions of the long arm. The nucleolus organizer regions showed a typical location in a pair of microchromosomes that exhibited Ag-NOR.The results obtained for *Nyctibius griseus* suggest that, of all the species studied in the references cited, it has the most ancestral sex chromosome composition of the order Caprimulgiformes.

## Introduction

At present, studies of the class Aves, which includes more than 9000 species, are fairly incomplete in regard to genetic and evolutionary studies (Pigozzi and Solari 2000). Cytogenetic studies have been conducted on less than 14% of the species content (Santos and Gunski 2006).

The species studied here, *Nyctibius griseus* (Gmelin, 1789) belongs to the order Caprimulgiformes, which includes the families Caprimulgidae, Nyctibiidae, Steatornithidae, Podargidae and Aegothelidae. *Nyctibius griseus* is found in South American territories from Costa Rica to Bolivia, Argentina, Uruguay and throughout Brazil, where it is common at the edges of forests, in fields with trees and on savannas. It feeds on nocturnal insects, mainly large moths, termites and beetles which it hunts in flight. It never lands on the ground, but always on branches, posts, fences and tree stumps, where it is easily camouflaged. This species has a form of adaptation unique among birds, known as the “magic eye” and consisting of two slits in the upper eyelid, which allows it to remain immobile for lengthy periods, watching its surroundings, even with its eyes closed. It lays one egg in a tree stump or branch cavity a few meters above ground level, where it is incubated for around 33 days (Accioly 2000). It is predominantly brownish in color, varying in tone from reddish to grayish, with streaks on its head and black markings on its breast. Its song consists of descending notes in the range of the human voice (Souza 2004).

Bird karyotypes generally consist of a diploid number of around 80 chromosomes, including eight macrochromosome pairs and 32 microchromosome pairs (Tegelström et al. 1983, De Lucca and Rocha 1992). According to Gunski et al. (2000), in species with a high number of microchromosomes, macrochromosomes with monobrachial or acrocentric morphology are prevalent. However, in species with a low number of microchromosomes, macrochromosomes with a bibrachial morphology are predominant, suggesting a process of karyotypic evolution through translocations between macro and microchromosomes, and also centric fusions of macrochromosomes.

In birds, a ZZ/ZW system determines gender, the male being the homogametic sex (the two sex chromosomes are homologous) and the female the heterogametic sex (the two sex chromosomes differ in size and morphology).

The members of the family Caprimulgidae are the most well-known of these birds, since they are found in all parts of the world, and consequently are better represented in terms of cytogenetic information. The references for the 40-year period of chromosome studies of this group of birds cited in Table 1 include descriptions of 8 karyotypes of Caprimulgidae: *Caprimulgus aegyptius arenicolor* (Lichtenstein, 1823), *Nyctidromus albicollis* (Gmelin, 1789), *Caprimulgus indicus* (Latham, 1790), *Hydropsalis brasiliana* (Gmelin, 1789*)*, *Chordeiles pusillus* (Gould, 1861), *Caprimulgus parvulus* (Gould, 1837) and *Caprimulgus rufus* (Boddaert, 1783) and *Lurocalis semitorquatus* (Gmelin, 1789). Two families of the five are represented by one species each – Podargidae, *Podargus strigoides* (Latham, 1801) and Nyctibiidae, *Nyctibius griseus*. There were no previous description of the karyotypes in the latter family, and the aim of this work was to show some details characterizing the chromosome complement of this New World species and establish the C- and Ag-NOR-banding patterns which may be evolutionary informative for these birds.

**Table 1.** Diploid number and morphology of macrochromosomes (in the authors’original transcription) in Caprimulgiformes species already karyotyped.

**Family/species**	**2n**	**Autosome pair number**													**Sex chromosomes**		**References**
		**1**	**2**	**3**	**4**	**5**	**6**	**7**	**8**	**9**	**10**	**11**	**12**	**13**	**Z**	**W**	
**Podargidae**																	
*Podargus strigoides*	72	SM	T	T	T	T	T	T	T	T	T	T	T	T	_	_	Belterman and De Boer 1984
**Caprimulgidae**																	
*Nictidromus albicollis*	78	ST	ST	ST	ST	ST	ST	SM	T	T	T	T	T	T	M	M	De Lucca and Waldrigues 1986
*Hidropsalis brasiliana*	74	T	A	T	A	A	SM	SM	M	SM	M	T	T	T	A	M	Nieto and Gunski 1998
*Chordeiles pusillus*	68	A	T	T	SM	T	T	A	M	A	T	_	_	_	SM	T	Nieto and Gunski 1998
*Caprimilgus aegyptius arenicolor*	70	ST	A	A	A	A	SM	SM	ST	A	A	_	_	_	SM	_	Bulatova et al. 1971
*Caprimilgus indicus*	76	ST	T	T	ST	T	M	M	T	T	T	_	_	_	M	_	Bian et al. 1988
*Caprimulgus parvulus*	72	M	SM	SM	SM	M	M	SM	M	M	M	_	_	_	SM	T	Nieto and Gunski 1998
*Caprimulgus rufus*	78	A	A	T	A	A	A	A	M	A	A	_	_	_	M	M	Nieto and Gunski 1998
*Lurocalis semitorquatus*	82	SM	ST	T	ST	ST	SM	M	M	SM	M	SM	T	M	_	_	Francisco et al. 2006
**Nyctibiidae**																	
**s*Nyctibus griseu***	**86**	**ST**	**SM**	**A**	**A**	**SM**	**SM**	**ST**	**M**	**M**	**A**	**A**	**A**	**A**	**SM**	**SM**	this study

2n = diploid number, M = metacentric, SM = submetacentric, ST = subtelocentric, T = telocentric, A = acrocentric.

## Methods

Specimens were captured from dusk to nightfall, the period of greatest activity, using nets set up over tree stumps and branches of trees in the Misiones Province, Campo San Juan (Sta. Ana), Argentina.

Two specimens were analyzed, 1 male and 1 female. They were taxonomically identified by Professor Julio Contreras. The specimens were deposited at Bernardino Rivadavia Natural Sciences Museum Collection, under accession numbers 011578 (male) and 011577 (female).

Metaphases were obtained using the direct bone marrow culture technique (Garnero and Gunski 2000). The constitutive heterochromatin was identified using a modification of the method described by Ledesma et al. (2002) and the karyotypes were arranged according to the classification in Levan et al. (1964). The nucleolus organizer regions (AgNOR) were determined according to the silver–nitrate method described by Howell and Black (1980).

## Results and discussion

**Figure 1.**
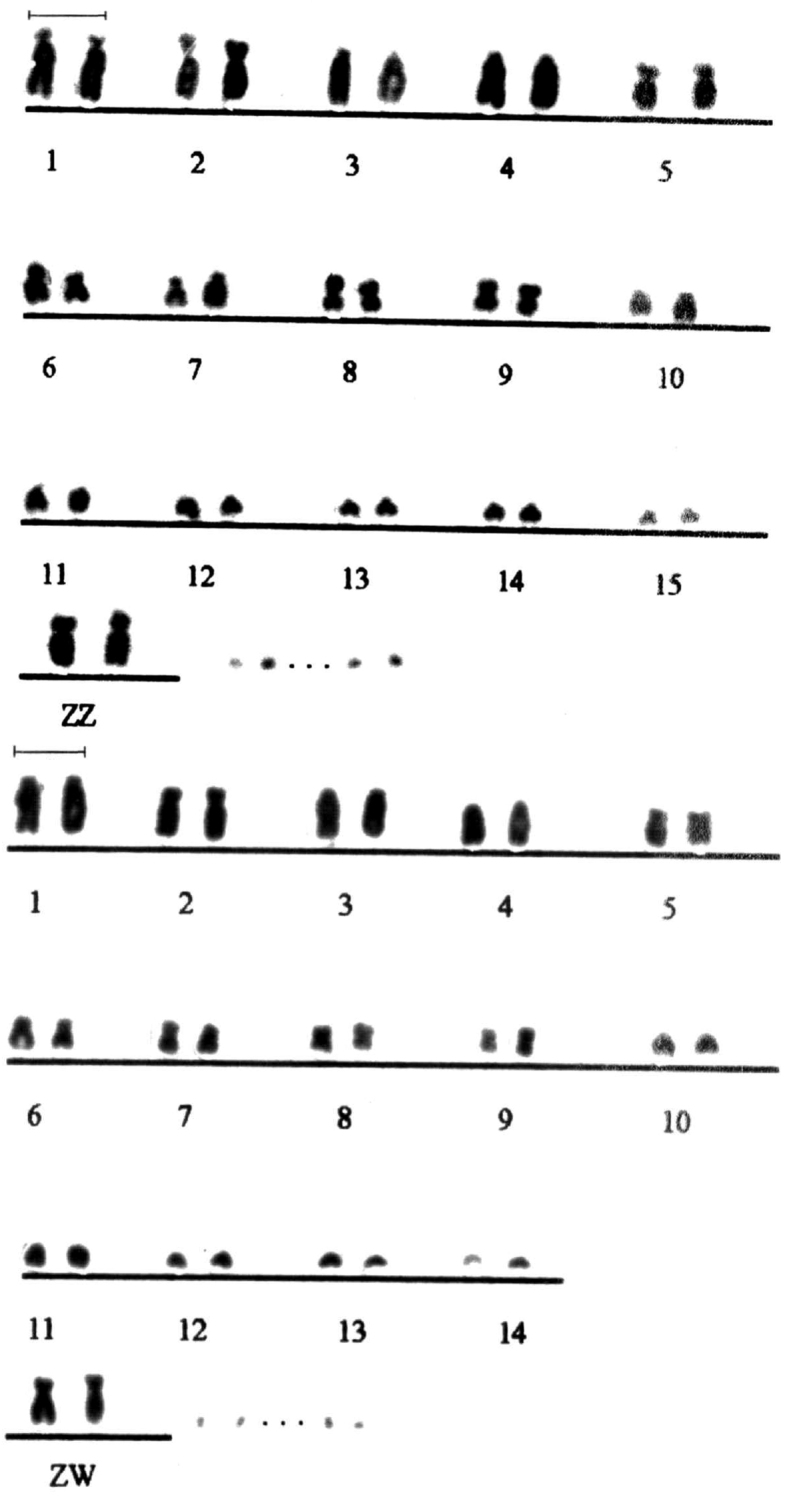
Male and female partial karyograms (without microchromosomes) of *Nyctibius griseus* (2n=86 ±). The similarity of the sex chromosomes ZZ and ZW is noticeable. Bar = 5 μm.

**Figures 2 A–D.**
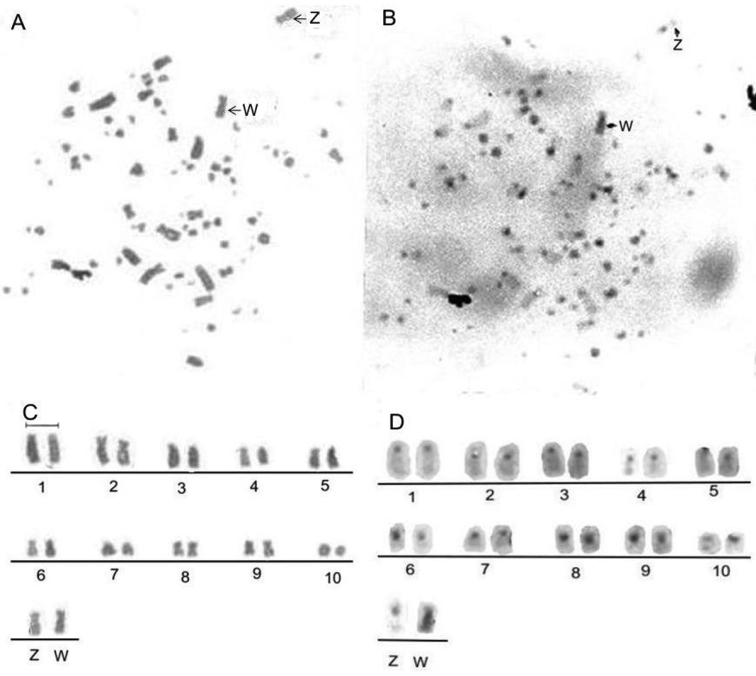
Metaphases and partial karyotype of female *Nyctibius griseus*: Giemsa (**A, C**) and C-banding (**B, D**) sequential staining. The arrows indicate the Z and W heterochromosomes. Among the autosomes, pairs 8 and 9 reveal an entirely heterochromatic short arm. Bar = 5 μm.

**Figure 3.**
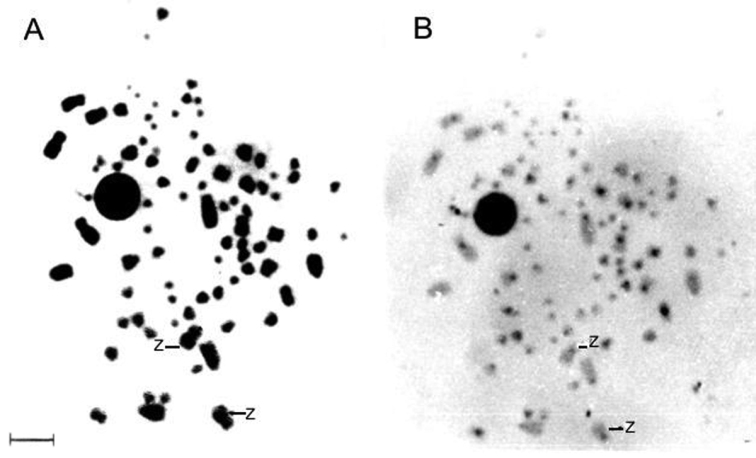
Routine Giemsa (**A**) and C-heterochromatin (**B**) sequential staining for a male *Nyctibius griseus*. The arrows indicate ZZ sex chromosomes. Bar = 5 μm.

**Figure 4.**
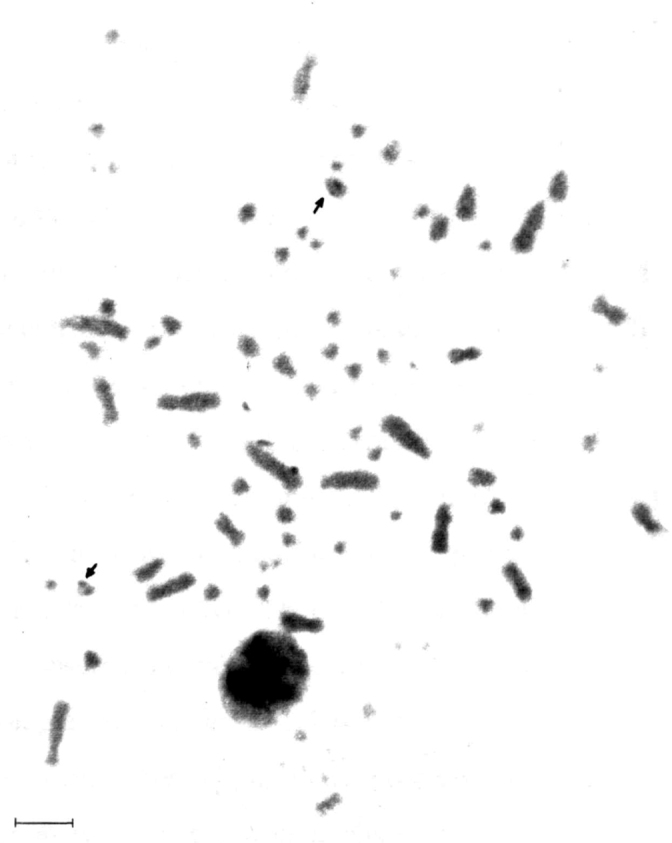
Nucleolus organizer regions in *Nyctibius griseus*. The arrows indicate NORs positioned in a strong secondary constriction probably of one pair of small one armed autosomes. Bar = 5 μm.

This study presents the initial data on the number and morphology of chromosomes of *Nyctibius griseus* (Figs 1–4). The diploid number in specimens of both sexes is 2n=86±. The first chromosome pairs are the large subtelocentric, submetacentric, acrocentric and acrocentric, and the following 5 pairs reveal bibrachial constitution (submeta-, subtelo- or metacentrics). Lesser chromosomes look mainly acrocentrics. The sex chromosomes of this species are interesting, since the W chromosome has metacentric morphology and size similar to the Z chromosome, so externally the ZZ pair in a male and ZW in a female (Fig. 1) look the same.

Using Giemsa/C-banding re-staining, we determined that, in contrast to the C-negative Z chromosome with a centromeric C-band, the W chromosome (Fig. 2) looks entirely C-band positive with the two most prominent markers in the interstitial and distal region of the long arm, something that is not observed when the chromosome has a higher degree of condensation. The C-banding pattern shows that all macrochromosomes exhibit a centromeric C-band, except for pairs 8 and 9 that have an entirely C-band positive arm.

Figure 3 shows the Giemsa C-banding sequential staining for a male *Nyctibius griseus*, highlighting the positive C-band in the centromeric region of the two Z chromosomes. The nucleolus organizer regions (Fig. 4) show up in a microchromosome pair that exhibits a strong Ag-NOR-positive band, as it is common in many birds.

8 species belonging to the family Caprimulgidae (Table 1) exhibit a marked numeric variability, ranging from 2n=68 in *Chordeiles pusillus* to 2n=82 in *Lurocalis semitorquatus*. Thus, the newly described karyotype of *Nyctibius griseus* with its 86 chromosomes shows the highest 2n for the whole order Caprimulgiformes. Without data on chromosome homology, any suggestion on karyotypic rearrangements is unreliable, nevertheless, a monotonous size arrangement of the karyotype without sharp differences between one- and bi-brachial chromosomes does not support a proposition of fusion between macrochromosomes. Thus, the 2n variation observed might result from fusions of microchromosomes to macrochromosomes. The morphology and large size of the W chromosome of *Nyctibius griseus* represents one of the most important discoveries in this study, and would lead us to infer that the species may be at a primitive stage of sex chromosome differentiation.

In the majority of bird species cytogenetically analyzed, the W chromosome is generally of a lesser size, close to the ninth or tenth pair, although there are cases like the one described by Christidis (1986) for *Neochmia phaeton* (Hombron & Jacquinot, 1841),a species in which the W chromosome is the third pair and is larger than the Z chromosome. Furthermore, in some species of Columbiformes (De Lucca and Aguiar 1976), Falconiformes (De Boer and Sinoo 1984), Passeriformes (Bulatova 1973) and Strigiformes (Renzoni and Vegni-Talluri 1966), the W chromosome was observed to be as large as the Z chromosome.

The W chromosome morphology in the family is also variable, ranging from metacentric to telocentric (Table 1). The wide variation in size and morphology of the W chromosome indicates different stages of differentiation, which shows that it has undergone greater changes in bird karyotype evolution than the Z chromosome.

In evolutionary terms, *Nyctibius griseus* may represent the first step in this differentiation, which according to Jones (1983), started from a homomorphic pair that acquired constitutive heterochromatin to become subsequently morphologically differentiated. These assumptions are in line with the distinct levels of heterochromatinization of the W chromosome of *Nyctibius griseus*, in which there is a general pattern of positive markers in the centromeric region, as well as an interstitial band and a telomeric region in the long arm.

The results we obtained for *Nyctibius griseus* lead us to assume that, of all the other Caprimulgiformes species studied, *Nyctibius griseus* exhibits the most ancestral sex chromsome composition.
